# Prevalence of Prostate Cancer at Different Clinical Stages in Italy: Estimated Burden of Disease Based on a Modelling Study

**DOI:** 10.3390/biology10030210

**Published:** 2021-03-10

**Authors:** Federico Spandonaro, Daniela D’Angela, Barbara Polistena, Paolo Bruzzi, Roberto Iacovelli, Irene Luccarini, Paola Stagni, Alessia Brigido

**Affiliations:** 1 Department of Economic and Finance, C.R.E.A. Sanità (Consortium for Applied Economic Research in Healthcare), University of Rome Tor Vergata, 00196 Rome, Italy; federico.spandonaro@uniroma2.it (F.S.); daniela.d.angela@uniroma2.it (D.D.); barbara.polistena@uniroma2.it (B.P.); 2 Unit of Clinical Epidemiology, Ospedale Policlinico San Martino—IRCCS, 16132 Genoa, Italy; bruzzipaolo49@gmail.com; 3 Medical Oncology Unit, Fondazione Policlinico A, Gemelli IRCCS, 00168 Rome, Italy; roberto.iacovelli@policlinicogemelli.it; 4 Janssen Cilag SpA, Cologno Monzese, 20093 Milan, Italy; pstagni@its.jnj.com (P.S.); abrigido@its.jnj.com (A.B.)

**Keywords:** disease progression, epidemiology, Italy, prevalence, prostatic neoplasms

## Abstract

**Simple Summary:**

The burden of prostate cancer is particularly high in Italy, where the disease ranks 4th among incident cancers overall and is 1st in men. In order to adequately plan health services and targeted interventions for the management of this neoplasm in Italy, it is of utmost importance to have a clear picture of the current number of patients surviving prostate cancer and of their needs in terms of cancer care. In our research, we aim therefore to quantify alive prostate cancer patients in Italy, and to provide relevant information on the number of survivors at different clinical stages of disease.

**Abstract:**

Understanding the distribution of prostate cancer (PC) at various clinical stages of disease is of utmost importance to quantify the cancer care needs of patients and to adequately plan health services. The aim of this analysis is thus to provide a model-based estimation of the number of prevalent PC patients at different clinical stages in the Italian setting. A simulation model of patient transitions was constructed on a yearly basis using data obtained through a literature review on the incidence, prevalence, progression and mortality of PC, with specific focus on disease stage. A total of 462,570 prevalent PC patients were estimated at 1 January 2019. According to the model, 94.8% of them had non-metastatic PC and 5.2% had metastatic disease. Among the non-metastatic patients, most had T1/T2 PC (85.6%), followed by T3/T4 (10.9%) and T0/Tx PC (3.6%). About 20% of the T3/T4 patients had biochemically recurrent PC. Among the metastatic PC patients, 66.1% had castration-resistant PC and 33.9% had hormone-sensitive PC. This study provided original information on the distribution of PC according to different clinical stages that may be useful to define strategies, understand the PC disease pathway, estimate treatment-related needs and, possibly, plan targeted interventions for public health management of prostate cancer in Italy.

## 1. Introduction

Over 1,250,000 new cases of prostate cancer (PC) are diagnosed every year worldwide, making this disease the 4th most frequent neoplasm in both sexes and the 2nd overall in men, after lung cancer [1]. Similar rankings are reported in the USA, where almost 192,000 incident PC cases are expected for the year 2020 (3rd incident cancer overall and 1st in men) [2], and in Italy, where the new diagnoses in 2020 should exceed 36,000 PC cases (4th incident cancer overall and 1st in men) [3]. PC is mainly a disease of elderly men and its diagnosis increases almost exponentially after the age of 50 years [4]. Since Italy is one of the world countries with the highest life expectancy at birth—82.8 years in both sexes and 80.8 years in men [5]—the number of PC patients is particularly high in Italy, being estimated around 450,000 patients in year 2018 (i.e., about 1.5% of the Italian male population).

Survival from PC improved over time, increasing from 65% (5-year survival) in the early 1990s to 91% during 2005–2009 in Italy [6]. This is explained by the higher detection of low-grade, low-risk PC after the introduction of PSA testing and by advances in disease characterization and treatment [7,8]. Noteworthy, an increased penetration of PSA screening can produce a dramatic increase in the prevalence of these low-grade PCs, due to their long preclinical detectable phase, and to their long survival after detection [9]. Further, steroid receptors play a pivotal role in PC, and many hormonal therapies targeting the androgen receptor signaling axis have been developed, increasing patient survival [7,8,9,10]. PC management differs widely according to stage of disease, with low-grade localized neoplasms often being indolent and harmless—thus needing active surveillance only—and more aggressive, advanced or metastatic disease requiring a range of interventions and treatments [10,11]. Understanding the distribution of PC at various clinical stages of disease is, therefore, of utmost importance to quantify the cancer care needs of patients and to adequately plan health services.

A number of modelling analyses were conducted in various world areas to examine the burden of PC at different stages [12,13], but to our knowledge no such investigation is available in Italy—a country with a high burden of disease. The aim of this analysis is thus to provide a model-based estimation of the number of PC patients at different clinical stages in the Italian setting.

## 2. Materials and Methods

In September 2019, we reviewed the international literature published from 2000 onwards through Medline, Embase, Web Of Science and Google Scholar, searching for data on the incidence, prevalence, progression and mortality of PC, with specific focus on disease stage. The bibliographic search started from the main pivotal trials of PC treatment that, through a “snowball method” approach, led to identify various robust studies with transferable data to inform the model. When more than one eligible investigation was found, the most recent one was used.

A simulation model of patient transitions, keeping into consideration the different stages of disease, duration of stay in each clinical stage and death, was constructed on a yearly basis using such data. Starting from yearly incident PC cases at different stages, we simulated disease progression and projected the cohorts, through literature-based estimates of mortality and transition between clinical stages, to the end of year 2018. In fact, an open-group model approach was used, considering 14 consecutive cohorts of incident PCs and implementing transitions on a yearly-basis. After reviewing the mortality data, it was observed that cohorts of patients diagnosed before 2004 gave a negligible contribution to the current burden of PC cases. Those cohorts were thus not considered in the models.

**Figure 1:**
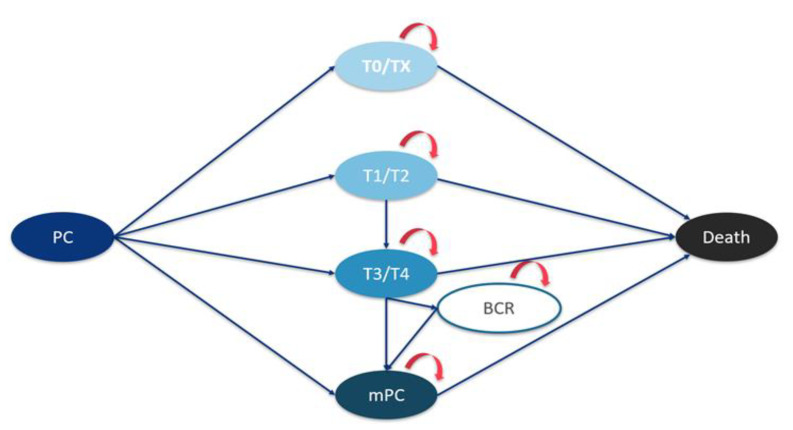
Diagram of the transition model.

Data on the number of incident PCs in Italy during the period from 2004 to 2018 were computed using incidence rates taken from the joint annual report of the Italian Association of Medical Oncology (AIOM) and Italian Association of Cancer Registries (AIRTUM) [6], combined with the annual Italian male population taken from the National Institute of Statistics (ISTAT) [14]. Since no population data on PC stage at diagnosis in Italy are available, data from neighbouring France—derived from the *Institut de Veille Sanitaire* (InVS), computed within the national program of *Surveillance épidémiologique des cancers en France*—were used to estimate the number of incident cases at each PC clinical stage [15]. According to the latter source, around 3% of incident cases are T0/Tx stage PCs, 27% are T1 stage, 58% are T2 stage PCs, 3% are locally advanced PCs and 10% are metastatic PCs. Thus, the following PC stages were defined: (i) initial PC (stages T0/Tx); (ii) localized PC (stages T1/T2); (iii) locally advanced PC (stages T3/T4); and (iv) metastatic PC. Additional stages considered in the model estimations were non-metastatic, biochemically recurrent (BCR) PC; metastatic, hormone-sensitive PC (mHSPC); metastatic, castration-resistant PC (mCRPC); and death. The prevalent cases were all subjects alive after a diagnosis of PC, with no time restrictions (i.e., including long-term survivors). The full structure of the model used to estimate the prevalence of PC at different clinical stages is reported in Figure 1.

Various studies provided information on the probability of transition from a clinical stage to another one, or to death [16,17,18,19,20,21,22,23]. These are reported, together with the corresponding probabilities, in Table 1. Overall mortality of the Italian population aged 65 years (assumed as mean age at PC diagnosis) in 2017—the most recent information available at the time of modeling—was used as background mortality [24]. Since scanty information is available, BCR-PC patients could not be further split into non-metastatic, hormone-sensitive PC and non-metastatic, castration-resistant PC.

In the above-reported sources, punctual information on mortality or progression of disease was extracted from the Kaplan–Meier curves using DIGITIZEiT v.2.3.3 software (DigitizeIt, Braunschweig, Germany); when needed, the data were then interpolated using SPSS v.23.0 (IBM, Armonk, NY, USA) statistical software.

Internal validation of the model was performed by comparing our estimated total prevalence of the PC cases in Italy in 2018 to the AIOM-AIRTUM data [6].

## 3. Results

Incident PC cases, computed on the basis of the AIOM-AIRTUM and ISTAT data, increased from 42,580 new cases in 2004 to a peak of 42,992 new cases in 2007, and then declined gradually to the lowest of 35,300 incident PC cases in the year 2018 (data not shown).

**Figure 2:**
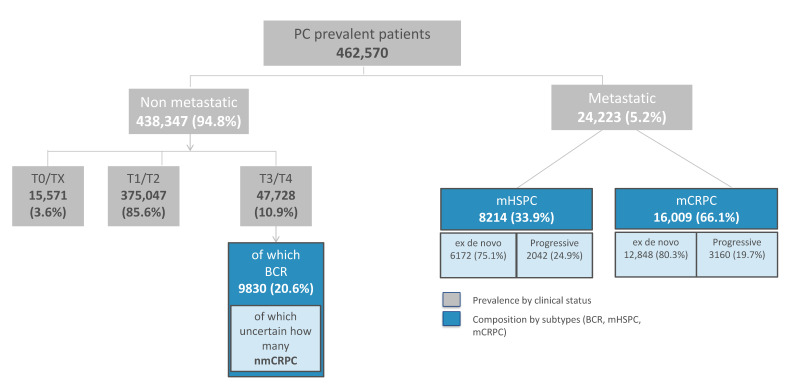
Estimation of the prevalent prostate cancer (PC) cases (according to their clinical stage) in Italy at 1 January 2019.

The estimated number of prevalent cases of PC in Italy at 1 January 2019 is shown in Figure 2, overall as well as according to the different clinical stages of PC. A total of 462,570 patients with a previous diagnosis of PC were estimated alive at the beginning of 2019. According to the model, 438,347 (94.8%) of them had non-metastatic PC and 24,223 (5.2%) had metastatic disease.

Out of 438,347 non-metastatic patients, the vast majority had T1/T2 PC (375,047; 85.6%), followed by T3/T4 PC (47,728; 10.9%) and T0/Tx PC (15,571; 3.6%). The model further estimated that 9830 of 47,728 (20.6%) T3/T4 patients had BCR-PC (including both non-metastatic recurrent CRPC and HSPC).

With reference to metastatic PC, mCRPC accounted for 66.1% of cases (*n* = 16,009, of which 12,848 (80.3%) were de novo metastatic PCs and 3160 (19.7%) were progressive metastatic PCs) and mHSPC contributed the remaining 33.9% of cases (*n* = 8214, of which 6172 (75.1%) were de novo metastatic PCs and 2042 (24.9%) were progressive metastatic PCs).

When compared to the AIOM-AIRTUM data, the model showed optimal performances as the total estimated number of prevalent cases was 462,570 vs. 458,000 (+1%), as reported by AIOM-AIRTUM [6].

## 4. Discussion

This analysis provided for the first time a model-based prevalence of PC cases at different clinical stages in Italy, estimating that about 85% of patients have early stage PC, 10% have advanced disease and 5% have metastatic disease. The final estimate of a total of about 460,000 alive patients surviving PC in 2018 is highly consistent with data provided by the AIOM-AIRTUM report—a national source relying on over 50 cancer registries throughout Italy—for the same year. This is supportive of the modeling process used in this study and thus on the general reliability of the results.

Two earlier analyses conducted in the USA and Australia modeled patient flow in PC. The first study [12] considered the national US incidence rates together with progression and mortality rates derived from original studies and meta-analyses to estimate, through a dynamic transition model, the existing and future burden of PC at 8 different clinical states. This investigation reported a proportion of metastatic PC cases of 4.4% in the year 2009 and 3.8% (projected) in 2020. The second analysis [13] was conducted in New South Wales, Australia, using cancer registry information (through an illness–death model) and hospital data on the treatment to estimate PC prevalence overall and according to subgroups of patients at different phases of care. The study estimated the progression to metastatic disease in 2.6% of patients in 2017, in addition to 2.1% of patients expected to die from PC during the same year. Thus, our data on metastatic PC (5.2%) are broadly consistent with those of the US and Australian studies.

A number of considerations related to our findings are required if these are to be used for planning cancer care services. In fact, the healthcare needs of cancer patients drastically change during the course of the disease, with maximum needs at diagnosis and at disease relapse or progression, and limited needs for long-term remitting survivors, which are often unrelated or partially related to the original cancer diagnosis. Most patients in our analysis had localized PC and, in consideration of the low incidence of disease progression in these subjects after surgery and/or radiation therapy [25], the majority of them are likely to be in disease remission. Long-term survivors also were included in the models, and most of them are likely definitively cured of the disease. Our model, however, did not allow to quantify the proportion of long-term PC remitting survivors. On the other hand, we provided an estimate of the number of prevalent T3/T4 PC cases, i.e., patients at high risk and with high probability of disease progression. This is particularly useful to adequately inform the understanding of the PC disease pathway, also in relation to the recent availability of new treatments, and potentially the design of future clinical trials in PC. We extracted data for the transition model from a set of identified references based on the standard of care. Still, since the frequency of prevalent PC depends—among other factors—from the efficacy of available treatments, the introduction in use of new therapies may lead to variations in the real number of prevalent patients. Further, for selected clinical stages—specifically for BCR PC—the heterogeneity in the natural history of disease, in its duration and treatment, is particularly high, thus making data modeling difficult. In fact, we could not divide and provide separate estimates for non-metastatic HSPC and non-metastatic CRPC, also in consideration of the small number of publications with useful data for the model.

A limitation of this study is related to the use of many international rather than national data to inform the model, due to the lack of specific studies from Italy. However, whenever possible we adopted estimates from countries close to Italy or with a similar setting, e.g., France. Several probabilities of transition, further, were extracted from international clinical trials conducted with a standardized methodology, and no major differences would be expected in studies from Italy alone. Another limit is that the prevalence estimates for low- vs. high-volume mHSPC were not available. For the latter, trial data suggest that about 55% are high-volume mHSPC [26]. Furthermore, androgen deprivation therapy is associated to increased risk of cardiovascular adverse effects [27], and patient comorbidities (particularly cardiovascular ones) may be a relevant issue for overall survival. However, data on the proportion of patients with comorbidities at different clinical stages of PC could not be considered in the model. The strengths of this investigation are its originality, as the study fills a gap of information in Italy—a country with a high incidence and prevalence of PC (1st neoplasm in men)—and the consistency of the estimated prevalence in PC between our and the AIOM-AIRTUM data, providing reassurance on the reliability of the model. Further, our model is based on a large set of published references, and the results may be relevant not only to Italy but also to other selected Western countries with a similar health system and distribution of PC cases.

## 5. Conclusions

Our study provide original information on the distribution of PC according to different clinical stages, which may be useful to define strategies, understand the PC disease pathway, estimate treatment-related needs and, possibly, plan targeted interventions for public health management of PC in Italy. An adequate knowledge of the number of PC patients at each clinical stage may support the action plans of policymakers by allowing a clearer picture of the therapeutic needs in our population and of the related healthcare resources to be implemented.

## Figures and Tables

**Table 1:** Cumulative probabilities of death or transition between the prostate cancer clinical stages used in the model.

Years Since Diagnosis/Clinical Stage Entry	Overall Mortality at 65 Years [24]	T1/T2 Mortality [23]	T3/T4 Mortality [16]	MET Mortality [20]	Transition from T1/T2 to T3/T4 [18]	Transition from T3/T4 to MET [16]	Transition from T3/T4 to BCR [19]	Transition from BCR to MET [22]	Transition to mCRPC, MET Patients at Diagnosis [17]	Transition to mCRPC, Patients Progressed to MET [21]
1	0.83%	1.34%	0.83%	16.98%	6.36%	4.93%	10.95%	0.00%	49.67%	28.19%
2	1.75%	2.67%	1.75%	33.57%	8.43%	9.96%	15.87%	3.61%	59.97%	42.30%
3	2.77%	4.09%	3.68%	46.12%	10.22%	15.00%	20.79%	7.34%	70.27%	53.73%
4	3.92%	5.65%	6.66%	55.26%	11.70%	20.04%	25.71%	11.07%	80.57%	62.74%
5	5.16%	7.29%	9.74%	61.61%	12.90%	25.07%	30.64%	14.79%	90.87%	69.61%
6	6.53%	9.07%	12.95%	65.80%	13.80%	30.11%	35.56%	18.52%	100.00%	74.62%
7	8.07%	11.01%	16.32%	68.44%	14.40%	35.14%	40.48%	22.25%	100.00%	78.05%
8	9.82%	13.17%	19.91%	70.17%	14.72%	40.18%	45.40%	25.98%	100.00%	80.16%
9	11.81%	15.55%	23.73%	71.61%	14.73%	45.22%	50.32%	29.71%	100.00%	81.24%
10	14.13%	18.28%	27.88%	73.38%	14.80%	50.25%	55.25%	33.43%	100.00%	81.56%
11	16.77%	21.32%	32.36%	76.11%	14.80%	55.29%	60.17%	37.16%	100.00%	81.41%
12	19.76%	24.72%	37.19%	80.42%	14.80%	60.32%	65.09%	40.89%	100.00%	81.04%
13	23.14%	28.50%	42.40%	86.93%	14.80%	65.36%	70.01%	44.62%	100.00%	80.75%
14	26.92%	32.69%	48.01%	96.27%	16.11%	71.17%	49.61%	40.63%	20.00%	19.46%
15	31.27%	37.44%	54.20%	100.00%	16.18%	76.27%	53.16%	43.34%	20.00%	19.46%

BCR: biochemical recurrence; mCRPC: castration-resistant metastatic prostate cancer; MET: metastatic prostate cancer.

## Data Availability

We copy-paste two publicly available datasets from the Italian Institute of Statistics (ISTAT) that were used in the models: other data to inform the models were taken from the scientific literature (http://dati.istat.it/), (http://demo.istat.it/) (accessed on 24 July 2020).

## References

[1] IARC-WHO (2018). GLOBOCAN. https://gco.iarc.fr/.

[2] American Cancer Society (ACS) Key Statistics for Prostate Cancer. https://www.cancer.org/cancer/prostate-cancer/about/key-statistics.html.

[3] AIOM (2020). I Numeri del Cancro in Italia.

[4] Haas G.P., Sakr W.A. (1997). Epidemiology of prostate cancer. CA Cancer J. Clin..

[5] WHO (2016). Global Health Observatory (GHO) Data. https://www.who.int/gho/mortality_burden_disease/life_tables/situation_trends_life_expectancy/en/.

[6] AIOM (2018). I Numeri del Cancro in Italia.

[7] Litwin M.S., Tan H.J. (2017). The Diagnosis and Treatment of Prostate Cancer: A Review. JAMA.

[8] Ilic D., Neuberger M.M., Djulbegovic M., Dahm P. (2013). Screening for prostate cancer. Cochrane Database Syst. Rev..

[9] Wilt T.J., Scardino P.T., Carlsson S.V., Basch E. (2014). Prostate-specific antigen screening in prostate cancer: Perspectives on the evidence. J. Natl. Cancer Inst..

[10] Attard G., Parker C., Eeles R.A., Schroder F., Tomlins S.A., Tannock I., Drake C.G., de Bono J.S. (2016). Prostate cancer. Lancet.

[11] Sartor O., de Bono J.S. (2018). Metastatic Prostate Cancer. N. Engl. J. Med..

[12] Scher H.I., Solo K., Valant J., Todd M.B., Mehra M. (2015). Prevalence of Prostate Cancer Clinical States and Mortality in the United States: Estimates Using a Dynamic Progression Model. PLoS ONE.

[13] Yu X.Q., Luo Q., Smith D.P., Clements M.S., Patel M.I., O’Connell D.L. (2017). Phase of care prevalence for prostate cancer in New South Wales, Australia: A population-based modelling study. PLoS ONE.

[14] ISTAT Demografia in Cifre. http://demo.istat.it/.

[15] Debrè M.B. Le Dépistage et le Traitement du Cancer de la Prostate. Annexe au Procès-Verbal de la Séance du 2 Avril 2009, No. 318, Sénat de France, Session Ordinaire de 2008–2009. http://www.senat.fr/notice-rapport/2008/r08-318-notice.html.

[16] Bolla M., Van Tienhoven G., Warde P., Dubois J.B., Mirimanoff R.O., Storme G., Bernier J., Kuten A., Sternberg C., Billiet I. (2010). External irradiation with or without long-term androgen suppression for prostate cancer with high metastatic risk: 10-year results of an EORTC randomised study. Lancet Oncol..

[17] Chi K.N., Agarwal N., Bjartell A., Chung B.H., Pereira de Santana Gomes A.J., Given R., Juarez Soto A., Merseburger A.S., Ozguroglu M., Uemura H. (2019). Apalutamide for Metastatic, Castration-Sensitive Prostate Cancer. N. Engl. J. Med..

[18] Eastham J.A., Kattan M.W., Fearn P., Fisher G., Berney D.M., Oliver T., Foster C.S., Moller H., Reuter V., Cuzick J. (2008). Local progression among men with conservatively treated localized prostate cancer: Results from the Transatlantic Prostate Group. Eur. Urol..

[19] Mason M.D., Parulekar W.R., Sydes M.R., Brundage M., Kirkbride P., Gospodarowicz M., Cowan R., Kostashuk E.C., Anderson J., Swanson G. (2015). Final Report of the Intergroup Randomized Study of Combined Androgen-Deprivation Therapy Plus Radiotherapy Versus Androgen-Deprivation Therapy Alone in Locally Advanced Prostate Cancer. J. Clin. Oncol..

[20] Pinsky P.F., Black A., Daugherty S.E., Hoover R., Parnes H., Smith Z.L., Eggener S., Andriole G.L., Berndt S.I. (2019). Metastatic prostate cancer at diagnosis and through progression in the Prostate, Lung, Colorectal, and Ovarian Cancer Screening Trial. Cancer.

[21] Tamada S., Iguchi T., Kato M., Asakawa J., Kita K., Yasuda S., Yamasaki T., Matsuoka Y., Yamaguchi K., Matsumura K. (2018). Time to progression to castration-resistant prostate cancer after commencing combined androgen blockade for advanced hormone-sensitive prostate cancer. Oncotarget.

[22] Tilki D., Preisser F., Graefen M., Huland H., Pompe R.S. (2019). External Validation of the European Association of Urology Biochemical Recurrence Risk Groups to Predict Metastasis and Mortality After Radical Prostatectomy in a European Cohort. Eur. Urol..

[23] Wilt T.J., Jones K.M., Barry M.J., Andriole G.L., Culkin D., Wheeler T., Aronson W.J., Brawer M.K. (2017). Follow-up of Prostatectomy versus Observation for Early Prostate Cancer. N. Engl. J. Med..

[24] ISTAT (2017). Tavole di Mortalità. http://dati.istat.it/.

[25] Hamdy F.C., Donovan J.L., Lane J.A., Mason M., Metcalfe C., Holding P., Davis M., Peters T.J., Turner E.L., Martin R.M. (2016). 10-Year Outcomes after Monitoring, Surgery, or Radiotherapy for Localized Prostate Cancer. N. Engl. J. Med..

[26] Hoyle A.P., Ali A., James N.D., Cook A., Parker C.C., de Bono J.S., Attard G., Chowdhury S., Cross W.R., Dearnaley D.P. (2019). Abiraterone in “High-“ and “Low-risk” Metastatic Hormone-sensitive Prostate Cancer. Eur. Urol..

[27] Muniyan S., Xi L., Datta K., Das A., Teply B.A., Batra S.K., Kukreja R.C. (2020). Cardiovascular risks and toxicity—The Achilles heel of androgen deprivation therapy in prostate cancer patients. Biochim. Biophys. Acta Rev. Cancer.

